# Research on Locally Resonant Characteristics of Pipelines with Periodic Structure

**DOI:** 10.3390/mi13060850

**Published:** 2022-05-29

**Authors:** Xingsheng Lao, Yonghua Yu, Fan Zhang, Jinxiao Ye, Xiangxi Xu, Zhaowang Xia

**Affiliations:** 1 Science and Technology on Thermal Energy and Power Laboratory, Wuhan 2nd Ship Design and Research Institute, Wuhan 430000, China; enginefluid@163.com; 2 School of Energy and Power, Jiangsu University of Science and Technology, Zhenjiang 212003, China; y15755586470@163.com (Y.Y.); yjxatpool@outlook.com (J.Y.); asluzw0820@163.com (X.X.); 3 Research and Development Center, China Academy of Launch Vehicle Technology, Beijing 100076, China; zhang316fan@163.com; 4 School of Shipbuilding and Ocean Engineering, Jiangsu Maritime Institute, Nanjing 211000, China

**Keywords:** local-area, phononic crystals, band gap, offshore platform

## Abstract

In this paper, the propagation characteristics of vibration waves in periodic pipelines were studied based on the band theory of phononic crystals, and we analyzed the influence of the geometrical structure parameters on the band gap characteristics of pipelines. The results show that, by increasing the number of layers of local resonant structure, both the initial frequency and the cutoff frequency of the band gap moved towards the lower frequency, while the width of the system band gap increased by 35 Hz, and the damping effect increased by 18.3 dB. By changing the thickness of the wall of the pipeline system, the width of the system band gap increased by 20 Hz, and the damping effect increased by 9.1 dB. The maximum vibration isolation of the offshore platform truss based on the periodic structure can be up to 7.93 dB. Therefore, it is feasible to apply the local resonant periodic structure to the vibration control of a practical offshore platform.

## 1. Introduction

Offshore platforms are a critical infrastructure for oil exploration worldwide [1,2]. Under the action of excitation loadings, such as winds, waves, earthquakes and ice, the offshore platform experiences large vibrations, which may be subjected to the destructive effects of fatigue failures. This will reduce personal comfortable feelings and endanger long-term structural health [3,4]. 

For high and medium frequency vibration control of offshore platform truss pipelines, technical means, such as laying viscoelastic damping materials in the piping are often used. These means may have some disadvantages, for example, the material is prone to aging. For the low frequency vibration, its energy is mainly concentrated in the low frequency band, which has the clear characteristic of a low spectrum line and is difficult to control [5,6].

Recently, the emergence of band gap properties of phononic crystal has injected vitality into the study of vibration control [7,8]. In the periodic structure, the band gap refers to the frequency band when the elastic wave is suppressed, and the frequency range of the band gap can be artificially adjusted [9,10,11]. Kaiming Bi [12] et al.’s innovative pipe-in-pipe system was proposed to mitigate subsea pipeline vibrations induced by various sources. Shen Huijie [13] introduced the periodic thought of phononic crystals into the design of pipes and calculated the bending vibration characteristics of periodic pipes with fluid-structure coupling by transfer matrix method. 

Yin Zhiyong introduced flexible nozzles into pipeline transverse vibration and analyzed the influence of flexible nozzles and pipeline support on vibration wave attenuation and propagation frequency band by solving transfer matrix characteristic values. Koo [14] et al. studied applying transverse periodic support to pipelines, calculated and analyzed the band gap of transverse bending vibration of pipelines and performed test verification, and found that transverse periodic support has certain vibration isolation effect on bending vibration. Therefore, the propagation of specific vibration wave by reasonable design of periodic structure can be controlled.

In this paper, phononic crystals are introduced into the design of the pipeline, and the finite-element analysis model of local resonant periodic pipeline structure is established. In the current paper, the damping performance of traditional pipeline structure and local resonance pipeline structures are compared, and the influence of the geometric structure parameters of the periodic local resonance pipeline on the initial frequency is investigated.

## 2. Theory and Mathematical Modeling

### 2.1. Computing Method of Band Gap

One primitive cell, which is in the infinite periodic spring–mass system, is simplified as a spring–mass system consisted of *n* springs connected with corresponding vibrator in series [15,16]. In this system, the gap between each spring vibrator is *l*, the size of the lattice constant is fixed as *h*. For a single periodic spring mass system, the dynamic equation of the *s* oscillator is calculated by Equation (1):

(1) $$m_{s}\ddot{x}=k_{s}(x_{s+1}-x_{s})-k_{s-1}(x_{s}-x_{s-1})$$

In the formula, s=2,3,⋯,n, *m_s_* is the mass of the mass block, *x_s_* is the displacement of the mass block, and *k_s_* is the stiffness of the material. Under the periodic boundary condition, the solution of Equation (1) can be written as Equation (2):

(2) $$x_{s}=A_{s}e^{i(q\sum_{s=2}^{s}d_{s}-\omega s)}$$

In the formula, *A_s_* is the amplitude of the vibrator *s*, ω is angular frequency, $q\sum_{s=2}^{s}d_{s}$ is the phase factor of the *s*th oscillator, *q* is the phase factor of the vibrator *s*, *d_s_* is the gap between vibrator *s* and vibrator *s* + 1; Wave vector *q_s_* is valued in the first Brillouin zone (−π/a, π/a), which $a=\sum_{s=1}^{n}d_{s}$.

By combining Equations (1) and (2), can find Equation (3):

(3) $$(k_{{}_{s}}+k_{{}_{s-1}}-\omega^{2}m_{s})A_{s}=k_{s}e^{iqd_{s+1}}A_{{}_{s+1}}+k_{{}_{s-1}}e^{-iqd_{s+1}}A_{{}_{s-1}}$$

The structure of the spring vibrators are periodical; therefore, we can acquire Equation (4) under the periodic boundary conditions.

(4) $$\left\{ \begin{array}{l} k_{0}=k_{n},k_{1}=k_{n+1}\\ m_{0}=m_{n},m_{1}=m_{n-1}\\ d_{0}=d_{n},d_{1}=d_{n+1}\\ A_{0}=A_{n},A_{1}=A_{n+1} \end{array}\right.$$

Coupling Equations (3) and (4), the matrices of linear equations are calculated by Equation (5):

(5) $$(Xq-\omega^{2}I)A=0$$

In the formula, $A={\left[A_{1},A_{2},\ldots A_{s}\right]}^{T}$, ***I*** is an n × n unit matrix. If ***A*** has a non-zero solution, its coefficient determinant equals zero; therefore, the solution of the Equation (5) transforms into the eigenvalue problem of n × n matrix ***X***. IN this way, we can determine the initial frequency and the width of band gap in the one-dimensional periodic structure.

### 2.2. Calculation Method of the Vibration Response

Equations (6)–(9) are the vibration equations of the pipeline in axial direction.

(6) $$f_{x}-GA_{p}k_{S}(\frac{\partial u_{x}}{\partial z})=0$$

(7) $$m_{y}-EI_{p}\frac{\partial \psi_{y}}{\partial z}=0$$

(8) $$\frac{\partial f_{x}}{\partial z}-(\rho_{p}A_{p}+\rho_{f}A_{f})\frac{\partial^{2}u_{x}}{\partial t^{2}}=0$$

(9) $$\frac{\partial m_{y}}{\partial z}+f_{x}-(\rho_{p}I_{p}+\rho_{f}I_{f})\frac{\partial^{2}\phi y}{\partial t^{2}}=0$$

In the formulas, *G* is the shear modulus of wall material, *f_x_, u_x_,* $\phi_{y}$ and *m_y_* are the shear force of the cross section of the pipeline in the *x* direction, displacement, turning corner and torque, respectively; ***I****_p_*, ***I**_f_*, ***A**_p_* and ***A**_f,_* respectively, are the cross-sectional inertia of the pipeline, the fluid cross-sectional inertia, the cross-sectional area of the pipeline, \ and the fluid cross-sectional area; and ρ is the material density of discrete element.

The radial direction vibration equation of a pipeline is:

(10) $$\rho \frac{\partial^{2}u}{\partial t^{2}}=\frac{\partial }{\partial x}(E\frac{\partial u}{\partial x})$$

In the formula, *u* refers to the displacement at *x*.

### 2.3. Evaluation Method of the Transfer Characteristics of Periodic Structure

Since the ideal phononic crystal is researched under the condition of infinite periodic structure; however, in practical engineering, the length of periodic structure is limited, therefore, we can only establish finite periodic structure in practical engineering application. The characteristic curve can used to judge if there are band gap, when the periodic pipeline under the external excitation.

If there is a band gap, note the initial and cut-off frequencies of the band gap. This paper used ANSYS to analyze and calculate the damping effect of periodic pipeline it is calculated by the harmonic response analysis module in ANSYS WORKBENC. The method is based on FEM and the mesh size is 0.05 m. The axial force excitation is applied at one end of the periodic pipeline structure, and the other end serves is used as the output response end, the acceleration vibration level different is used to evaluate the damping effect of the finite pipeline. The definition is as follows:

(11) $$L_{D}=20\mathrm{lg}\frac{a_{0}}{a}$$

In the formula, *a*_0_ is the output acceleration, *a* is the input acceleration, and the analysis frequency range in this paper is 0–1000 Hz.

### 2.4. Structural Modeling and Experimental of Local Resonant Periodic Pipeline

**Figure 1:**
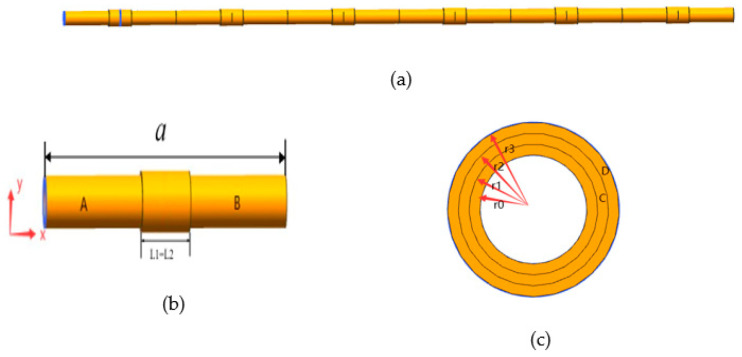
The periodic pipeline structure: (**a**) partly periodic structure; (**b**) single periodic structure; and (**c**) local oscillator cross section.

Figure 1a shows the composite periodic pipeline model, Figure 1b demonstrates the single periodic pipeline structure. The structure consists of an inner pipeline and two layers of outer pipelines, two layers of pipelines are nested on the inner pipeline to form a locally resonant periodic pipe. The inner pipeline consists of section A and section B. 

The material of section A is aluminum and the material of section B is steel. The outer local resonance structure is consisted of materials C and D. Material C is epoxy resin and D is steel. L1 is the width of material C, L2 is the width of material D. The length of material A and material B are same. The Inner diameter of inner pipeline is r_0_, the inner diameter of the first outer pipeline is r_1_, diameter of the second outer pipeline is r_2_, the thickness of material C is h1 = r2 − r1 and the thickness of material D is h2 = r3 − r2. The physical parameters of the material are shown in Table 1. The parameters for the cycle piping structure are a = 10 m, r_0_ = 0.6 m, r_1_ = 0.625 m, r_2_ = 0.635 m, r_3_ = 0.645 m, L_1_ = L_2_ = 2 m, h_1_ = 0.02 m, h_2_ = 0.02 m. There are six periods with a total length of 60 m.

**Figure 2:**
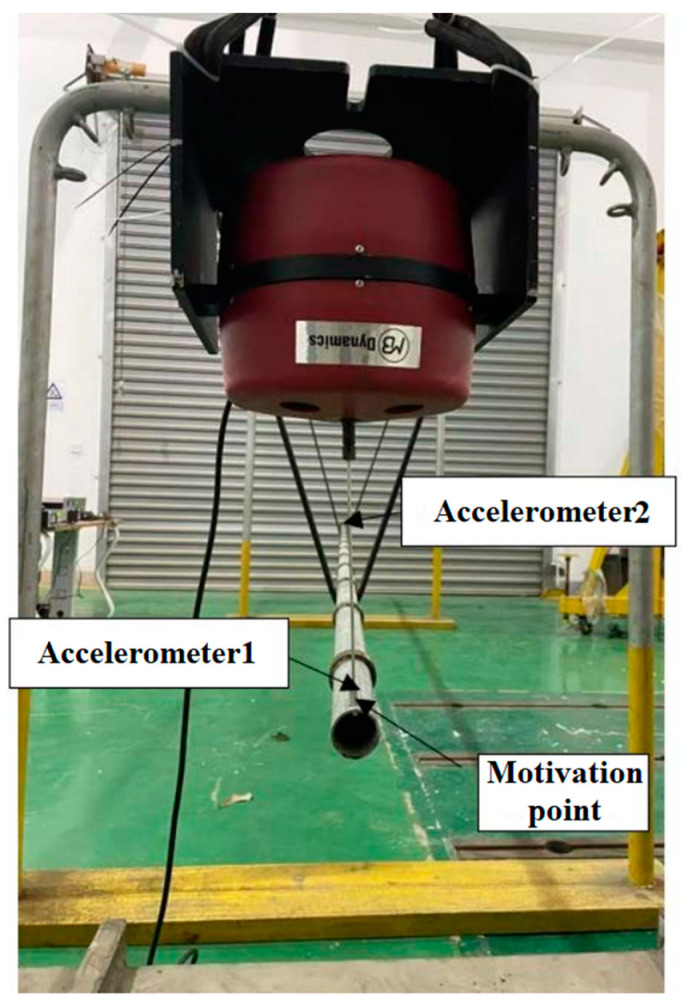
Vibration damping performance test platform with local oscillator cycle pipe.

A test system for the vibration level difference of common pipes and local oscillator periodic pipeline was built, as shown in Figure 2. The excitation point as shown in the figure. Experiment generates excitation through the exciter, it a harmonic excitation. The excitation step is 5 Hz and the frequency range is 10–1000 Hz, and the acceleration response positions are point 1 at the input end and point 2 at the output end. The pipeline system is supported by angle steel, and the support position is divided into the middle and both ends. 

In the test, common pipes is a pipe without local resonant layers, the model material of the local oscillator periodic pipeline is a galvanized pipe, with the pipeline length of 6000 mm, the outer diameter of 48 mm and the inner diameter of 42 mm. periodic pipe is a pipe with local resonant layers. On the basis of common pipes, we added 12 local resonant layers. The width of a single local resonant layer was 27 mm, the inner diameter was 48 mm, and the outer diameter was 62 mm. The pipe material parameters are shown in Table 2.

**Figure 3:**
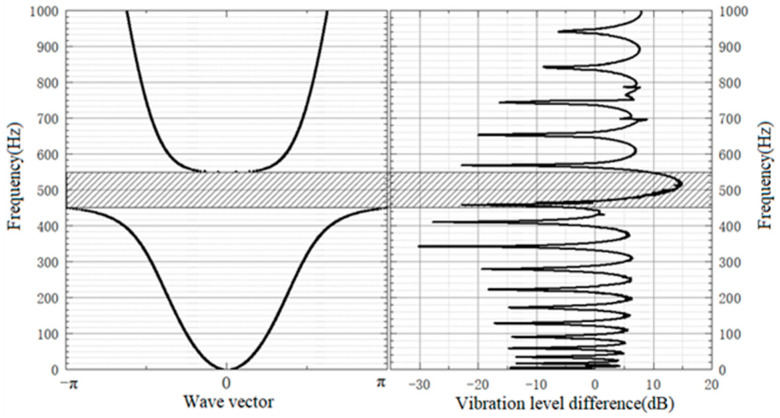
Comparison of energy band structure and vibration transmission characteristics of local oscillator period lines.

The vibration drop of local oscillator periodic pipeline is obtained by Siemens vibration noise analysis and processing as shown in Figure 3. The band structure in Figure 3 is calculated theoretically.

It can be seen from the results in Figure 3 that the band gap in the band structure of the local oscillator periodic pipeline is 461–552 Hz, and the band gap in the vibration transfer characteristic curve is 458–570 Hz, which has a good consistency, indicating that the band gap range of the periodic pipeline can be adjusted through the periodic structure design of the local oscillator. By comparing the experimental data with the theoretical data, it can be seen that the experimental results of the band gap characteristics of the ship pipelines with local oscillators are consistent with the theoretical results, which verifies the correctness of the theoretical calculation method.

## 3. Analysis of Band Gap Property

### 3.1. The Effects of the Local Resonant Layers on the Band Gap

**Figure 4:**
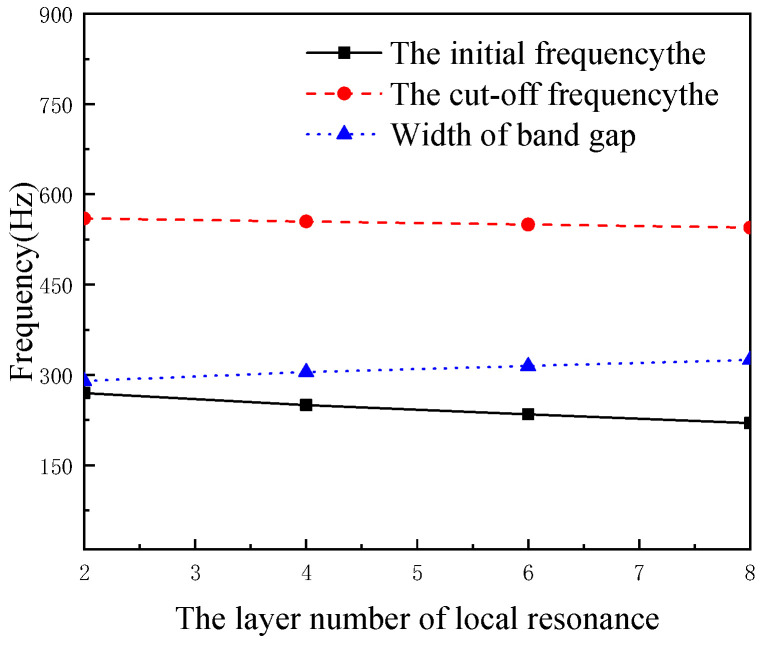
The effect of the local resonant layers on the band gap.

When keeping other parameters unchanged, we increase the number of local resonant layers and studied the influence of layer numbers band gap. The number of layers are 2, 4, 6 and 8 layers, respectively. The layers are distributed as epoxy resin + steel, epoxy resin + steel + epoxy resin + steel and so on. The effect of local resonant layers on the band gap is shown in Figure 4.

Figure 4 demonstrates that the initial frequency and the cut-off frequency of the bandgap are moving to lower frequency, with the initial frequency decreasing from 270 Hz to 220 Hz and the decreasing degree of the initial frequency being greater than the degree of the cut-off frequency. Therefore, the width of band gap increases from 290 Hz to 325 Hz.

**Figure 5:**
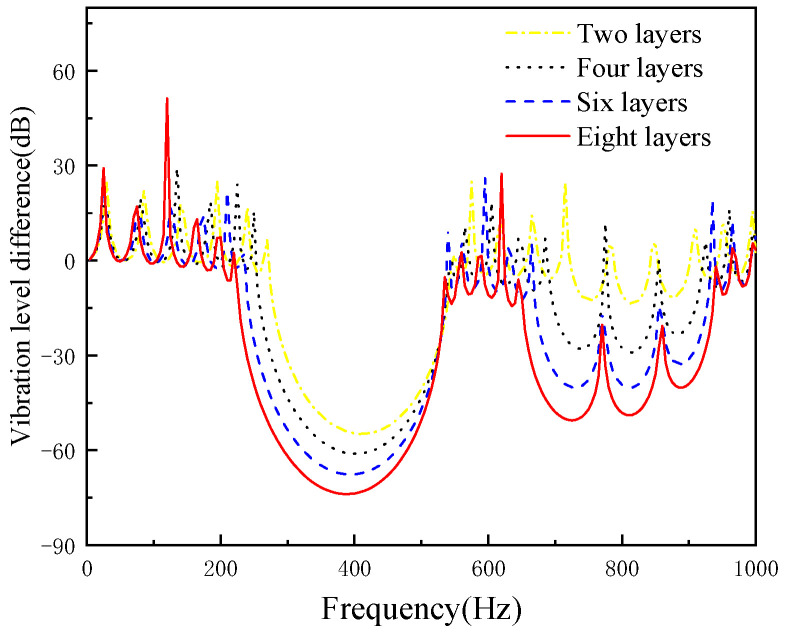
The effect of the local resonant layer number on the cushioning performance.

On the basis of period pipeline model of the phononic crystal, only the number of local resonant layers is changed and other parameters are unchanged, and the acceleration damping performance of the model is calculated when the number of local resonant layers is 2, 4, 6 and 8 layers, as shown in Figure 5. As can be seen from the figure, with the increase of the number of local resonant layers of the pipeline, the damping performance of the pipeline is becoming increasingly better, and the effect of damping is improved by 18.3 dB.

### 3.2. The Effects of the Local Resonance Wall Thickness on the Band Gap

**Figure 6:**
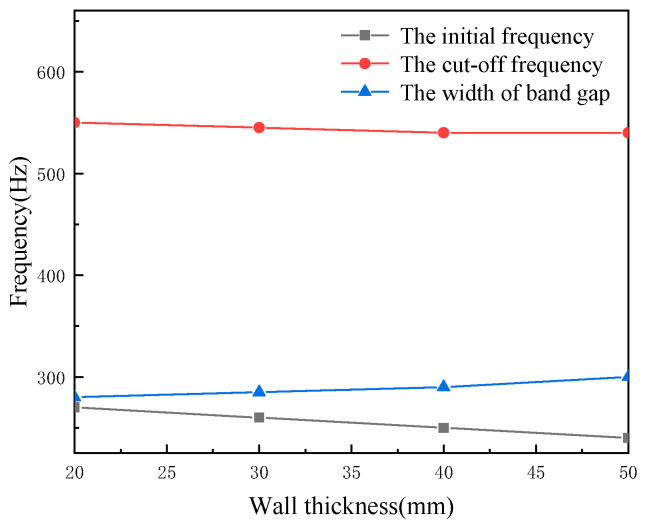
The effects of local resonant wall thickness on the band gap.

The material of pipeline A is aluminum, the material of pipeline B is steel. The local resonance is two layers, the inner layer material is epoxy resin and the outer layer material is steel. This section studied the effects of thickness of local resonance pipeline on the band gap characteristics, the wall thickness of the local resonance is 20, 30, 40 and 50 mm, and the results are shown in Figure 6. It is clear that as the thickness increasing from 20 mm to 50 mm, the initial frequency and the cut-off frequency is reducing, and the cut-off frequency attenuation factor of the band gap is lower than the initial frequency, and the width of first band gap increased by 300 Hz.

**Figure 7:**
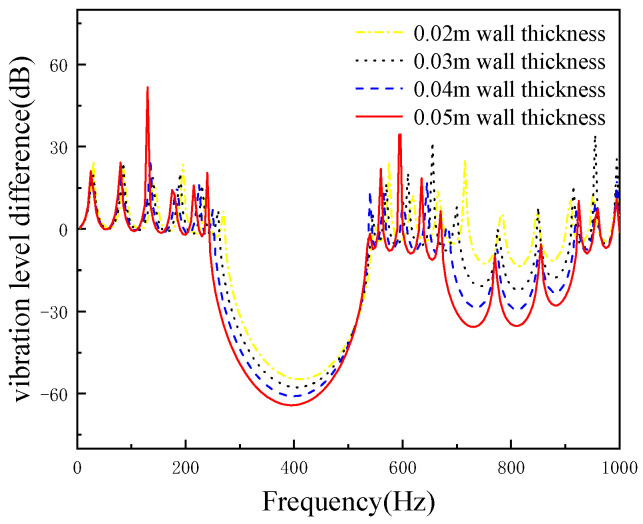
The effect of the wall thickness on the cushioning performance.

The lattice constant of periodic pipeline is 10 m, study the effects of local resonance unit wall thickness on vibration control. When the thickness of wall is 20, 30, 40 and 50 mm, respectively. The four cushioning performance curves of pipelines with different wall thickness are shown in Figure 7. It is seen that the four different wall thicknesses have the same number of band gaps, and the damping effect of the pipeline increases from 11.4 dB to 20.5 dB with the increase of wall thickness, which increases by 9.1 dB.

## 4. Analysis of Vibration Damping Performance

**Figure 8:**
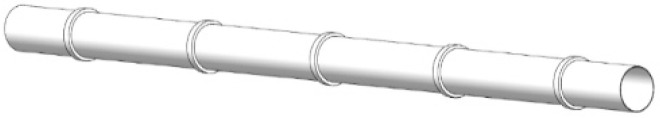
Finite period pipe structure.

**Figure 9:**
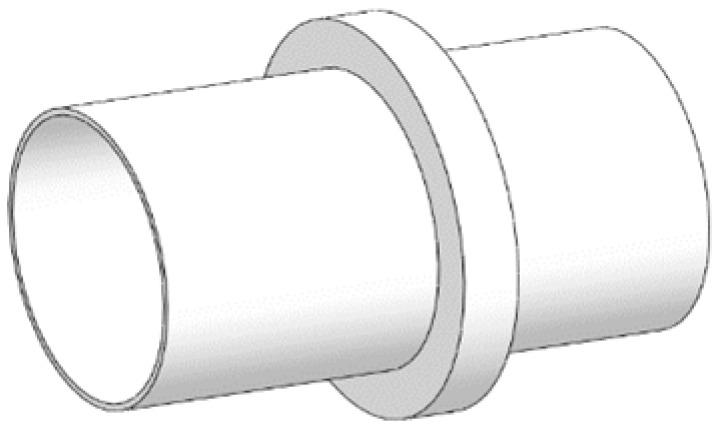
Single periodic structure.

The model material of Figure 1 is set as steel material, the local resonance is one layer, the material of pipe is steel, its density is 7850 kg/m^3^, the elastic modulus is 200 Gpa, and the Poisson ratio is 0.3. The finite periodic pipeline is shown in Figure 8, the inner diameter of pipe is 1.2 m, the outer diameter of pipe is 1.25 m, and the pipeline lattice constant is 10 m. The single periodic pipeline is shown in Figure 9. 

**Figure 10:**
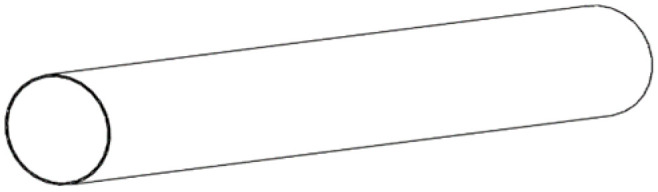
Common pipes.

Common pipes are shown in Figure 10. The height and width of the local resonance on the structure of the periodic pipeline are studied on the influence law of the vibration level drop, the height of the periodic structure is 10 mm, 20 mm, 30 mm and 40 mm, and the width of the periodic structure is 40 mm, 60 mm and 80 mm. The input vibration, output and total vibration difference in each working condition are shown in Table 3.

**Figure 11:**
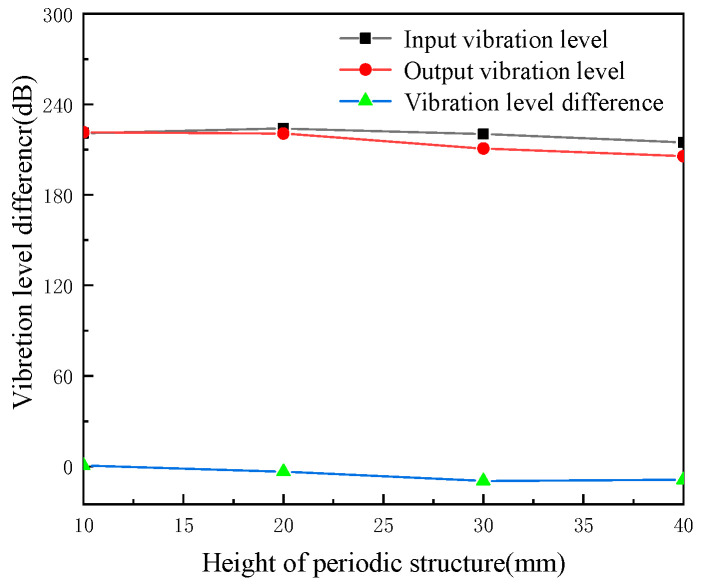
The effects of periodic structure height on cushioning performance (periodic structure width of 40 mm).

We keep the local resonance width of the periodic pipe structure 40 mm and other parameters unchanged. The vibration level at the input end, the vibration level at the output end and the vibration level drop of the pipeline are shown in Figure 11 when the resonance height of the period structure is 10 mm, 20 mm, 30 mm and 40 mm, respectively. From the figure, it can be seen that with increase of the local resonance height, the vibration level at the input and output of the pipeline decreases in turn, and the vibration level difference between them also reduces with the increase of height. The increase of height of the structure results in the reducing of the vibration level difference; thus, the vibration damping effect of the pipeline is weakened.

**Figure 12:**
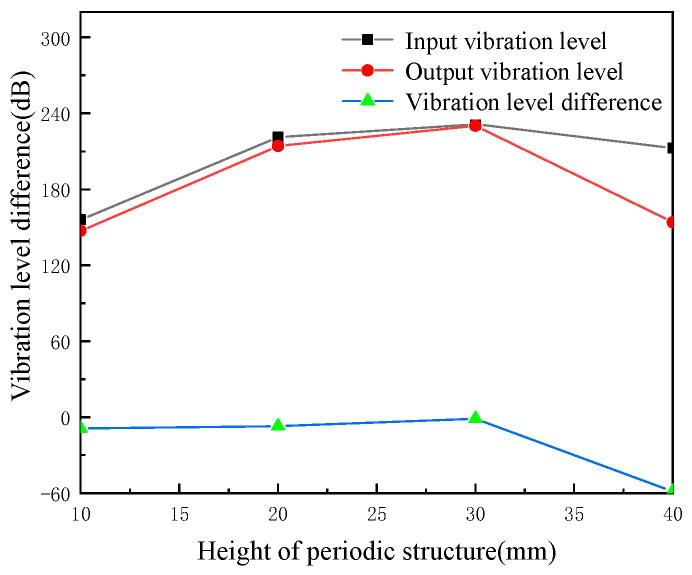
The effects of periodic structure height on cushioning performance (periodic structure width of 60 mm).

We maintained the width of the periodic pipeline structure local resonance at 60 mm and the other parameters as invariable. We studied the pipeline’s input and output vibration level and vibration level difference when the height of periodic structure local resonance is 10 mm, 20 mm, 30 mm, 40 mm, respectively, and the results are shown in Figure 12. 

When the width of local resonance is 60 mm, with the increase of local resonance height, the vibration level of the input end and output end of the pipeline first becomes larger and then decreases, when the height is 20 mm, the maximum vibration level of the input end of the pipeline reaches 218.40 dB. When the height is 40 mm, the vibration level of the output end of the pipeline is the smallest, 139.18 dB. The vibration level of the pipeline falls with the increase of the local resonance height, the maximum vibration level of the pipeline falls 12.36 dB. When the height is 40 mm, the maximum vibration level of the pipeline is 12.36 dB.

**Figure 13:**
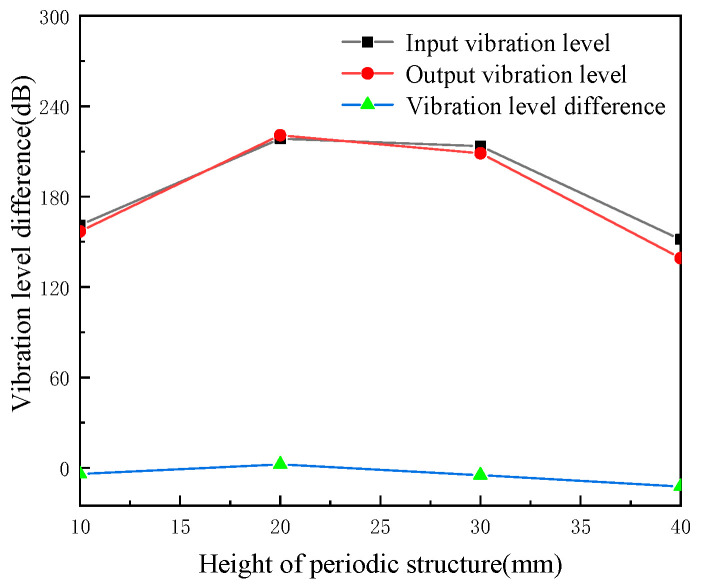
The effects of periodic structure height on cushioning performance (periodic structure width of 80 mm).

The width of the periodic structure is kept at 80 mm, and the influence of the height of the periodic structure on the vibration level at the input end, the vibration level at the output end and the vibration level difference is studied. The heights selected for the pipeline are 10, 20, 30 and 40 mm, and the results are shown in Figure 13. It can be seen from the figure that when the height of the periodic structure increases, the vibration level of the input end and the output end of the pipeline increases first and then decreases, and the vibration level difference decreases slowly first and then increases with the increase of the local resonance height. When the height is 40 mm, the vibration level difference reaches the maximum value of 58.6 dB.

**Figure 14:**
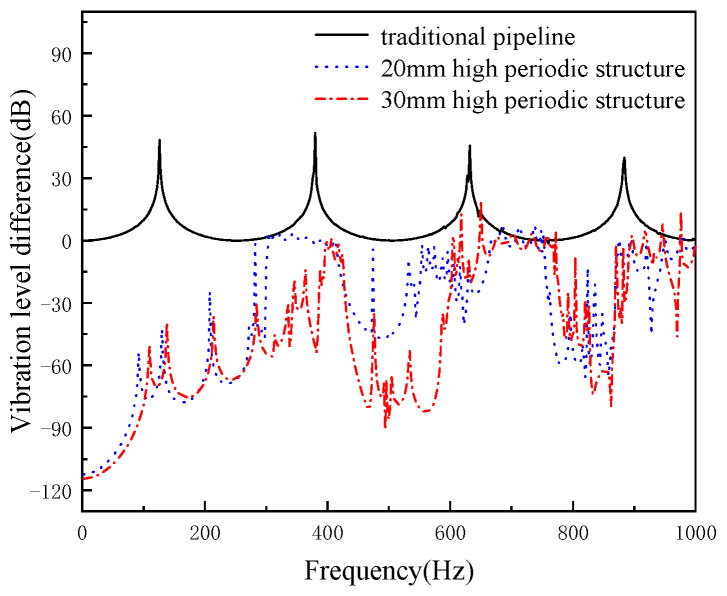
The effects of periodic structure height on cushioning performance (periodic structure width of 40 mm).

The width of the local resonance structure of the periodic pipeline is maintained at 40 mm, and the axial vibration reduction performance of the pipeline is shown in Figure 14 when the height of the periodic structure is 20 mm and 30 mm. It can be seen from the figure that when the local resonance width is 40 mm, when the periodic structure height is 20 mm and 30 mm, both of them have two broadband band gaps, while the traditional pipeline does not appear band gap. Increasing the height can effectively reduce the initial frequency and increase the cut-off frequency of the band gap, so as to obtain a wider band gap. Increasing the height can effectively increase the damping effect in the band gap.

## 5. Study on the Vibration Characteristics of the Periodic Pipeline Structure of the Offshore Platform

### 5.1. The Modeling the Offshore Platform

**Figure 15:**
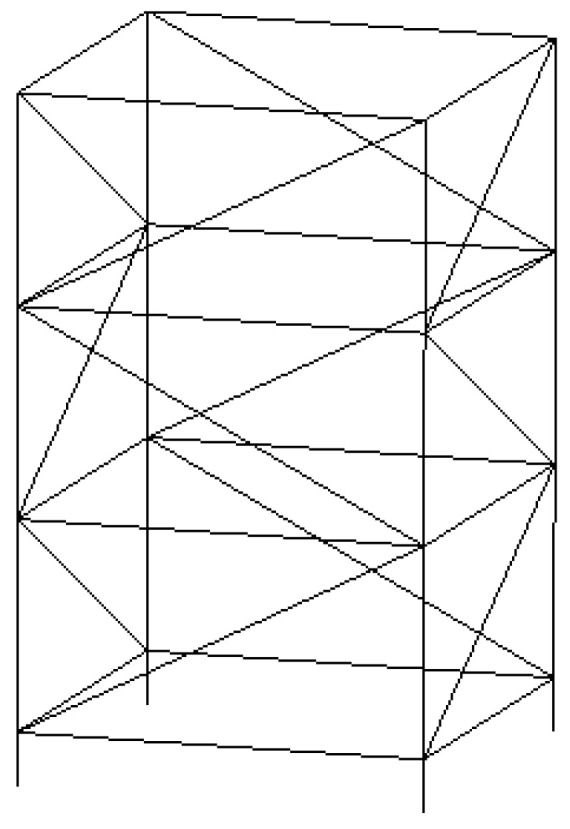
Offshore platform truss structure model.

A simplified model of the offshore truss platform is established as shown in Figure 15. It has 3 layers, the total height of the four main pipes is 65 m, each floor height is 20 m, and the height of fixed pillar at bottom is 5 m. The working platform at the top of the marine platform truss is 0.02 m thick steel plate. The related parameters of the offshore truss platform are shown in Table 4.

**Figure 16:**
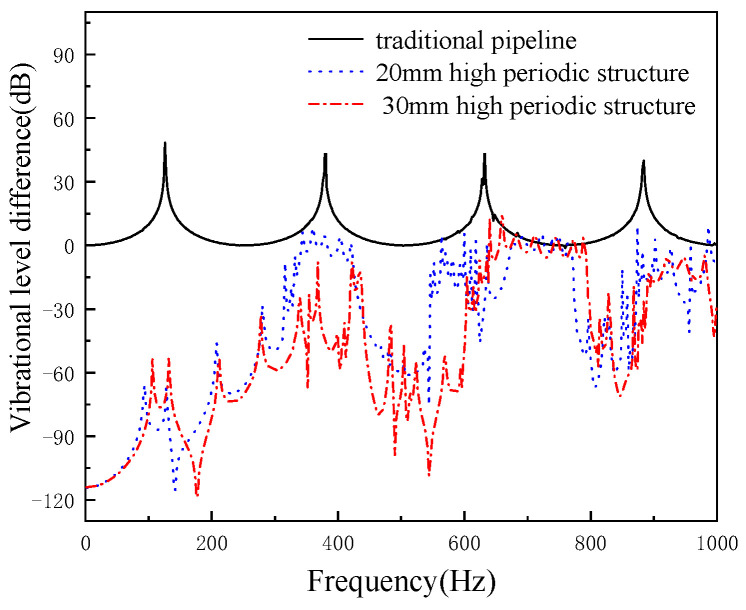
The effects of periodic structure height on cushioning performance (periodic structure width of 60 mm).

When the width of periodic pipeline local resonance is 60 mm, the height of structure is 20 mm and 30 mm, respectively, the axial cushioning performance of a pipeline is shown in Figure 16. It is shown that when the width is 60 mm, increasing the height of structure can shift the center frequency of the band gap to high frequencies, and the damping performance is also improved.

**Figure 17:**
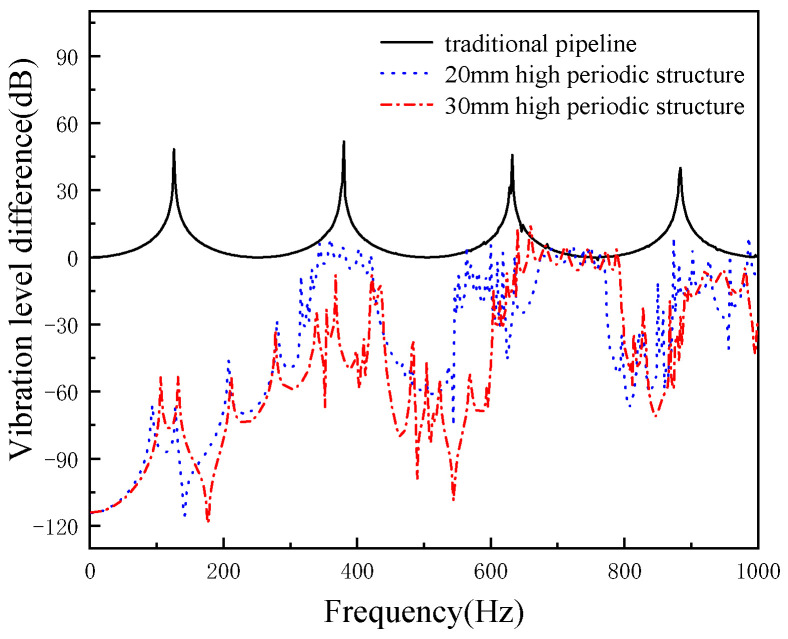
The effects of periodic structure height on cushioning performance (periodic structure width of 80 mm).

The axial damping performance of the pipeline when the width of the periodical pipeline local resonance structure is unchanged at 80 mm, and the height of the periodical structure is 20 mm and 30 mm, respectively is shown in Figure 17. It can be seen from Figure 17 that the height of the periodic structure is 20 mm and 30 mm, and there are two band gaps in the frequency range of 0–1000 Hz. With the increase of the height of the period structure, the damping effect of the pipeline is also improved.

### 5.2. Effect of Local Resonance Width on Damping Performance

Through the above research on band gap characteristics and vibration damping performance considered only local resonant cycle pipe, it is known that the cycle pipe has better vibration damping performance than the traditional pipeline. Therefore, in order to improve the vibration damping performance of the offshore truss platform, the inner diameter of marine platform truss cycle inner pipe is 1.2 m, outer diameters is 1.25 m metal steel. The outer pipe is 0.02 m height metal rigid, the lattice constant is 10 m, and the material physical parameters are shown in Table 1.

Keep other parameters invariable, study the effects of different periodic pipeline local resonance width on the offshore truss platform’s vibration level by increasing width. The widths are 10 mm, 20 mm, 30 mm, 40 mm, 50 mm, 60 mm, 70 mm, 80 mm, respectively.

**Figure 18:**
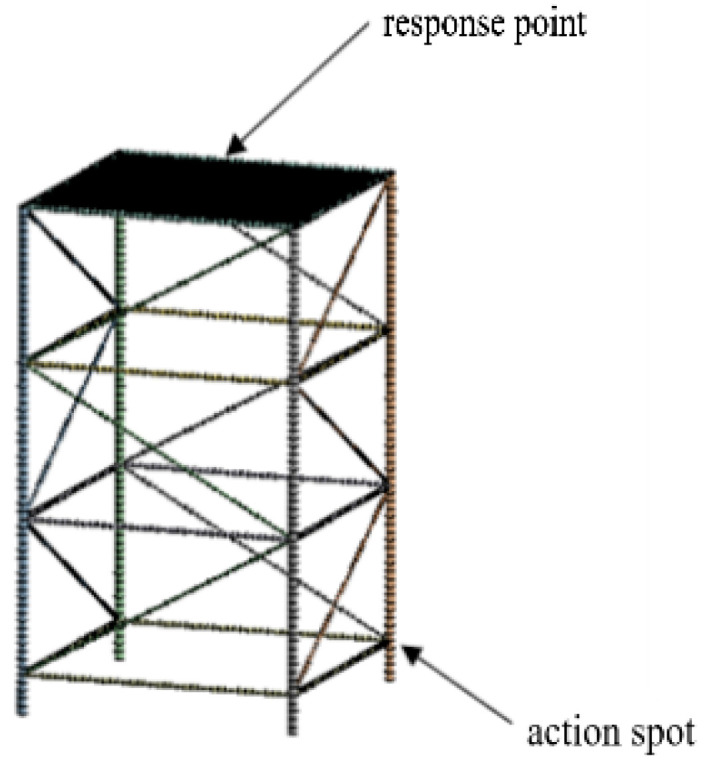
Offshore platform truss structure model.

**Figure 19:**
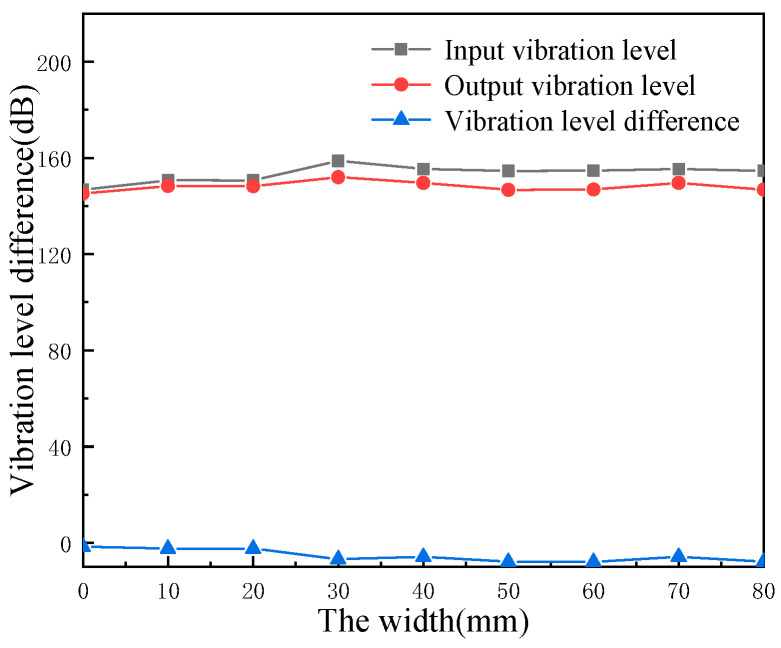
The effect of the local resonance width on the vibration level.

The axial excitation and radial excitation are applied at the intersection of the horizontal pipeline and the four main pipelines at the bottom of the ocean platform truss structure, and the excitation and response positions are shown in Figure 18. The acceleration response at the excitation and the acceleration response at the center of the upper panel are extracted, and the vibration level at the bottom and the vibration level at the top as well as the vibration level drop can be obtained after processing the data, and the obtained results are shown in Table 5 and Figure 19.

According to Figure 19, the cushioning performance of offshore periodic truss platform is better than that of traditional offshore platform. As the width of local resonance increases, the vibration level of the offshore periodic truss platform input terminal decreases in turn, the vibration level of output terminal increases first and then decreases. The vibration level difference increases first and then decreases, the maximum of vibration isolation can reach to 7.93 dB.

## 6. Conclusions

In this paper, the concept of a local resonant phononic crystal is introduced to the offshore platform pipeline to improve the cushioning performance, a finite-element analysis model of the periodic pipeline is established, the cushioning performance of a finite periodic structural pipeline is calculated, and the effects of the geometric structure parameters of the pipeline on the band gap are analyzed. We obtained the following rules:

(a) The geometry parameters of the periodic pipeline influence the band gap initial frequency, cut-off frequency and band gap width and clearly affect the pipeline vibration loss.
(b) Changing the number of layers and thickness of the local resonance allows the initial frequency of the band gap to move towards the low band while wide band gap is obtained.
(c) As the height of the pipeline increases, both the pipeline input and output vibration levels decrease in turn, and the vibration difference between the two also decreases with the increasing height.
(d) The cushioning performance of an offshore platform with a periodic truss structure is better than that of a traditional offshore platform. As the width of the local resonance increases, the vibration level of the offshore periodic truss platform input terminal decreases in turn, the vibration level of output terminal increases first and then decreases. The vibration level difference increases first and then decreases, and the maximum vibration isolation can reach 7.93 dB.

## Figures and Tables

**Table 1:** The physical parameters of the periodic pipeline material.

Material	Density (kg·m^−3^)	Modulus of Elasticity E (Gpa)	Poisson Ratio
steel	7850	200	0.3
aluminum	2700	70	0.33
Epoxy resin	1180	4.35	0.38

**Table 2:** The physical parameters of the periodic pipeline material.

Material	Density (kg·m^−3^)	Modulus of Elasticity E (Gpa)	Poisson Ratio
steel	7850	200	0.3

**Table 3:** The cushioning performance of periodic pipeline under different working conditions.

Height (mm)	Width (mm)	Input Terminal Vibration Level (dB)	Output Terminal Vibration Level (dB)	Vibration Level Difference (dB)
10	40	220.88	221.46	0.58
20	40	224.08	220.59	−3.49
30	40	220.33	210.70	−9.63
40	40	214.62	205.67	−8.95
10	60	161.16	156.99	−4.17
20	60	218.40	220.72	2.32
30	60	213.59	208.73	−4.86
40	60	151.54	139.18	−12.36
10	80	156.04	147.2	−8.84
20	80	221.14	214.15	−6.99
30	80	231.42	230.21	−1.21
40	80	212.61	154.02	−58.59
Traditional pipeline		241.06	240.91	−0.15

**Table 4:** Offshore platform pipeline parameters.

Material	Inner Diameter (m)	Outer Diameter (m)	Quantity
Main pipeline	1.2	1.25	4
Horizontal pipeline	0.66	0.7	16
Inclined pipeline	0.38	0.4	12

**Table 5:** The effect of local resonance width on vibration level.

Width (mm)	Input Terminal Vibration Level (dB)	Output Terminal Vibration Level (dB)	Vibration Level Difference (dB)
Traditional trusses	146.70	145.12	−1.58
10	150.72	148.32	−2.40
20	150.65	148.24	−2.41
30	158.76	152.02	−6.74
40	155.37	149.59	−5.78
50	154.61	146.74	−7.87
60	154.74	146.82	−7.93
70	155.37	147.59	−7.79
80	154.63	146.77	−7.86

## Data Availability

The data presented in this study are available on request from the corresponding author.
